# Systematic Analysis of RNA Regulatory Network in Rat Brain after Ischemic Stroke

**DOI:** 10.1155/2018/8354350

**Published:** 2018-01-08

**Authors:** Juan Liu, Kun-shan Zhang, Bin Hu, Si-guang Li, Qing Li, Yu-ping Luo, Yang Wang, Zhi-Feng Deng

**Affiliations:** ^1^Department of Neurosurgery, Shanghai Jiao Tong University Affiliated Sixth People's Hospital, Shanghai, China; ^2^Stem Cell Translational Research Center, Tongji Hospital, Tongji University School of Medicine, Shanghai, China; ^3^Institute of Microsurgery on Extremities, Shanghai Jiao Tong University Affiliated Sixth People's Hospital, Shanghai, China

## Abstract

Although extensive studies have identified large number of microRNAs (miRNAs) and long noncoding RNAs (lncRNAs) in ischemic stroke, the RNA regulation network response to focal ischemia remains poorly understood. In this study, we simultaneously interrogate the expression profiles of lncRNAs, miRNAs, and mRNAs changes during focal ischemia induced by transient middle cerebral artery occlusion. A set of 1924 novel lncRNAs were identified and may involve brain injury and DNA repair as revealed by coexpression network analysis. Furthermore, many short interspersed elements (SINE) mediated lncRNA:mRNA duplexes were identified, implying that lncRNAs mediate Staufen1-mediated mRNA decay (SMD) which may play a role during focal ischemia. Moreover, based on the competitive endogenous RNA (ceRNA) hypothesis, a stroke regulatory ceRNA network which reveals functional lncRNA:miRNA:mRNA interactions was revealed in ischemic stroke. In brief, this work reports a large number of novel lncRNAs responding to focal ischemia and constructs a systematic RNA regulation network which highlighted the role of ncRNAs in ischemic stroke.

## 1. Introduction

Stroke is the second leading cause of long-term disability in high-income countries and the second leading cause of death worldwide [1]. Numerous biological processes are regulated in the progression of ischemic stroke, ranging from deprivation of oxygen, neuron necrosis, to intense inflammatory response [2, 3]. Previous studies have discussed the RNA program involved in cerebral ischemia including miRNAs and lncRNAs [4–7], which contribute to RNA-mediated regulation network, but gap still remained in our knowledge of ischemic stroke. The RNA-mediated regulation network consists of many kinds of RNA molecules, such as miRNA, lncRNA, and circRNA. These noncoding RNAs (ncRNAs) regulated the important cellular events via variety mechanisms and have profound effects on the outcome of ischemic stroke [8, 9]. Understanding these precise RNA molecular mechanisms after cerebral ischemia on a system-wide level is critical for exploring potential new strategies for early diagnosis and therapy of stroke.

Long noncoding RNAs (lncRNAs), which are the majority products of mammalian genomes, were proved to be critical gene regulators of development and disease [10–12]. Recent studies showed that lncRNAs are heterogeneous noncoding RNAs with different regulatory mechanisms. The proposed mechanisms including transcriptional and epigenetic mechanisms via the recruitment of transcription factors and chromatin-modifying complexes to specific nuclear and genomic sites act as cis-regulatory elements or act in trans to modulate gene expression, modulation of chromatin architecture [12–14]. LncRNAs may also perform their function by competitively binding to miRNAs, which is known as competitive endogenous RNA (ceRNA) [15], or by mediating mRNA decay via Staufen1-mediated mRNA decay (SMD) [16] to regulate genes expression. Previous study has shown that cerebral lncRNAs were significantly altered after stroke and contribute to the stabilization of mRNA expression [6]. Stroke-induced lncRNAs can interact with chromatin-modifying proteins and modulated ischemic brain damage-related genes [17, 18]. Moreover, coding noncoding coexpression network analysis showed that expression of lncRNA BC088414 was correlated with apoptosis-related genes following hypoxic-ischemic brain damage. Silencing of BC088414 in PC12 cells decreased cell apoptosis and increased cell proliferation [7]. A very recent study found that lncRNA C2dat1 promoted neuronal survival by upregulated CaMKII*δ* expression following cerebral ischemia [9]. However, compared to mountains of RNA molecules (especially lncRNAs) identified via massive parallel sequencing, little is known about functional RNA molecules and RNA-mediated regulation network in ischemic stroke.

To reveal the RNA-mediated regulation network in rat ischemia cortex, we profiled cortex of rat transient middle cerebral artery occlusion model. We identified 2588 lncRNAs in the rat cortex, 1924 (~74%) of which were regarded as novel lncRNAs since they have not been annotated in the rat genome (rn5, Ensembl 2015). Ten novel lncRNAs were validated as significantly differentially expressed genes (*P* < 0.05, paired *t*-test) after 24 hours of reperfusion compared with the contralateral brain. Importantly, we identified several subsets of lncRNAs associated with biological processes during MCAO via coexpression network analysis. SMD-mediated RNA decay was analyzed by calculating thermodynamic features of lncRNA:mRNA duplex. Furthermore, we have constructed a systematic lncRNA-miRNA-mRNA network which reveals a complex functional RNA-mediated regulatory network in ischemic stroke. Our results, for the first time, described a global view of RNA-mediated regulation network in cerebral ischemia and shed light on discovering new functional regulators of ischemic stroke.

## 2. Materials and Methods

### 2.1. Animals

All animal procedures were approved by Ethics Committee of Shanghai Jiao Tong University and were performed according to the guidelines of the US Department of Health for use and care of laboratory animals.

### 2.2. MCAO Model and Tissue Preparation

The transient middle cerebral artery occlusion (MCAO) model in rat was performed as described previously [19]. Briefly, Sprague-Dawley male rats (weight 250–280 g) were intraperitoneally anesthetized with chloral hydrate (0.9 ml/100 g body weight). A 4-0 nylon suture with silicon was inserted into internal carotid artery through the incision of external carotid artery and gently advanced to occlude the middle cerebral artery. After 2 hours of MCAO, the suture was carefully removed to restore blood flow, and rats with score 2 according to Zea-Longa 5-point scale (0, no deficit; 1, failure to extend right paw; 2, circling to the right; 3, falling to the right; and 4, unable to walk spontaneously) were used in the following study.

Animals were sacrificed 24 h after MCAO and the brains were removed and sliced into 2 mm coronal sections (approximately +3.0 to −5.0 from bregma) using a brain matrix. The ipsilateral and contralateral tissues from the two middle slices (+1 to −3 from bregma) of MCAO subjected animals were used for subsequent RNA isolation, while the outer two slices were used to confirm infarct formation by staining with 2,3,5-triphenyltetrazolium chloride (TTC).

### 2.3. RNA-Seq

RNA-seq was performed at Shanghai BioChip Company (Shanghai, China). Briefly, total RNA of each sample was prepared using RNeasy Mini kit (QIAGEN, Germany). Libraries were constructed by standard TruSeq protocol. Purified cDNA libraries were used for cluster generation and sequenced on the Illumina HiSeq 2500 according to the manufacturer's protocol. After masking the adaptor sequences and the removal of contaminated reads, clean reads were processed for in silico analysis. The reads were mapped using TopHat with 2 mismatches allowed. The expression of RNA in brain was expressed in fragments per kilobase of exon per million reads mapped (FPKM) and calculated by the TopHat and Cufflinks package. The FASTQ files have been deposited in the NCBI GEO database under accession number GSE78200.

Small RNA-sequencing has been described previously [19]. Briefly, total RNA was prepared using the mirVana™ miRNA Isolation Kit (Ambion). 5′- and 3′-Adapters were ligated to the obtained small RNA. Reverse transcription followed by PCR was used to create cDNA constructs. Subsequently, a 145 nts to 162 nts fraction corresponding to approximately the adapter-ligated constructs derived from the 20 nts to 35 nts small RNA fragments was excised and purified. The purified libraries were sequenced on Illumina HiSeq 2000 apparatus. The raw data were refined using fastx (fastx_ toolkit-0.0.13.2). After filtering out low quality reads and short reads (<18 nts), the small RNA reads were compared to the Sanger miRBase database (http://www.mirbase.org/) to identify known miRNAs, and the unidentified sequences were further aligned to several other small RNA databases, including the ncRNA Database, piRNA Database, and Rfam Database. Small RNA-sequencing data was deposited on GEO (GSE70473).

### 2.4. LncRNAs Annotation Pipeline

Total reads were aligned to rat genome (Rn5) by TopHat (v2.01). Reference annotation based transcripts (RABT) were reconstructed by Cufflinks (v2.11) with [-b,-u] options and annotations from Ensembl (February 2015). Transcripts constructed from Cufflinks were compiled together by Cuffcompare. Transcripts detected in at least 5 (half of all) samples were considered as bona fide transcripts. Transcripts, except those with just one exon and short than 200 bp, were further analyzed for identification of lncRNAs. Transcripts with class code “i,” “r,” “u,” “x,” and “.” were selected as novel long transcripts. New transcripts were compared to other annotation databases including NONCODE (v4) (http://www.noncode.org), NCBI RefSeq, UCSC, and Ensembl. CPAT (v1.22) was used to estimate coding potential of each novel transcript. Transcripts with a CPAT score < 0.487 were considered as lack of coding potential and were subjected to a BLASTX search for similar protein sequences. Briefly, a set of 10,000 mRNA sequences and 10,000 randomly selected intron sequences were used as training dataset to estimate rat specific cutoff of CAPT score with comparing Ensembl coding genes by AUC analysis. 0.487 is selected as cutoff value since at this threshold the sensitivity and specificity are maximized. Transcripts that have no hits in BLASTX were accepted as new lncRNAs.

### 2.5. Conservation Analysis of LncRNAs

Two conservation algorithms were used to evaluate conservation of a given sequence: PhastCons and phyloP. Basewise genomic conservation profiles generated by PhastCons (ftp://hgdownload.cse.ucsc.edu/goldenPath/rn5/phastCons13way/) or phyloP (ftp://hgdownload.cse.ucsc.edu/goldenPath/rn5/phyloP13way/) for multiple alignments of 13 vertebrate genomes to rat genome were straightly downloaded from UCSC genome browser and used in our analysis. Basically, the phyloP/phastCon scores of transcripts were defined as the average phyloP/phastCon score of each nucleotide of its exons. Nucleotides which have no phyloP/phastCon score were ignored. To aid in discovery of putative ultraconserved elements and conserved transcripts, we applied two metrics: the fraction of significantly conserved bases (phyloP score > 2) and the maximum conserved 200 nt sliding window (mean PhastCons score of each window) as described by Iyer et al. [20].

### 2.6. Hub-Based Function Analysis of LncRNAs

The Pearson correlation coefficient (PCC) between lncRNAs and every mRNA detected in our dataset was calculated to construct a coding-noncoding gene coexpression networks. The *P* value of each PCC was estimated by Fisher's asymptotic distribution. A coexpression gene pair was defined by the adjusted *P* value < 0.01 and the value of PCC was in the top 0.01% or bottom 0.01% of each lncRNA. Function of each lncRNA was defined by GO term (biological process only) that is enriched in coexpressed protein-coding genes of each lncRNA. According to Liao et al., a GO term that had a *P* value ≤ 0.01 (hypergeometric test) and genes ≥ 5 was accepted [21].

### 2.7. Short Interspersed Elements (SINE) Distribution Analysis

3′UTR sequences of rat mRNAs were downloaded from UCSC genome browser. LncRNAs sequences and mRNA 3′UTR sequences were analyzed by RepeatMasker (http://www.repeatmasker.org/cgi-bin/WEBRepeatMasker) to identify SINE elements. RNA_RNA_anneal was used to predict thermodynamically stable duplexes between lncRNAs and mRNA 3′UTR [16]. Δ*G* distribution was analyzed by “fitdist” function in “fitdistrplus” R package. *P* value of each Δ*G* datapoint was calculated and any duplex with *P* ≤ 0.05 was accepted.

### 2.8. Construction of LncRNA-miRNA-mRNA Networks

Genes (including lncRNAs, mRNAs, and miRNAs) that are differentially expressed between two conditions were taken into account. To construct lncRNA-miRNA interaction network, we used miRanda (v3.3a) to analyze all lncRNAs with default parameters. Interactions with miRanda score ≥ 150 and Δ*G* < −20 kcal/mol will be used as edges of network, and the corresponding miRNA and lncRNA will be used as node. The mRNA-miRNA network was constructed similarly: miRNA and its targeting mRNA will be used as node, and the interaction relationship as edge. Predicted miRNA binding sites in protein-coding genes were downloaded from http://www.microrna.org/microrna/getDownloads.do, including predicted interactions with good mirSVR score and conserved miR, predicted interactions with nongood mirSVR score and conserved miR, and predicted interactions with good mirSVR score and nonconserved miR. To restrict the size of miRNA-mRNA network, we calculate the Pearson correlation coefficient (PCC) between each pair of miRNA-mRNA, and the *P* value of each PCC was estimated by Fisher's asymptotic distribution. Only miRNA-mRNA that has negative PCC with *P* value ≤ 0.05 was subsumed by miRNA-mRNA interaction network. The lncRNA-miRNA network and miRNA-mRNA network were merged by the same node of miRNA.

### 2.9. Quantitative Real-Time PCR and RT-PCR

Total RNA were isolated using Trizol reagent (Invitrogen, Life Technology, USA), 1 ug RNA from each sample was reverse-transcribed into cDNA and subjected to quantitative real-time PCR (qRT-PCR), and RT-PCR. qRT-PCR was performed using the SYBR Green I Kit (Roche, Switzerland) according to the manufacturer's protocol. RT-PCR was performed with 2x Taq PCR MasterMix (TIANGEN Biotech, Beijing, China) at *T*_*m*_ as 60°C. GAPDH amplified with 25 PCR cycles were used as control, and all lncRNAs were amplified with 35 cycles. Primers used for PCR are summarized in Table 1.

### 2.10. Statistical Analyses

Statistical analysis of RNA-seq data was conducted as described above. Real-time PCR data were analyzed by analysis of variance followed by the least significant difference test. The data are shown in the figures as mean ± SD. *P* < 0.05 was considered significant.

## 3. Results

### 3.1. Identification of LncRNAs in Ischemic Stroke

**Figure 1:**
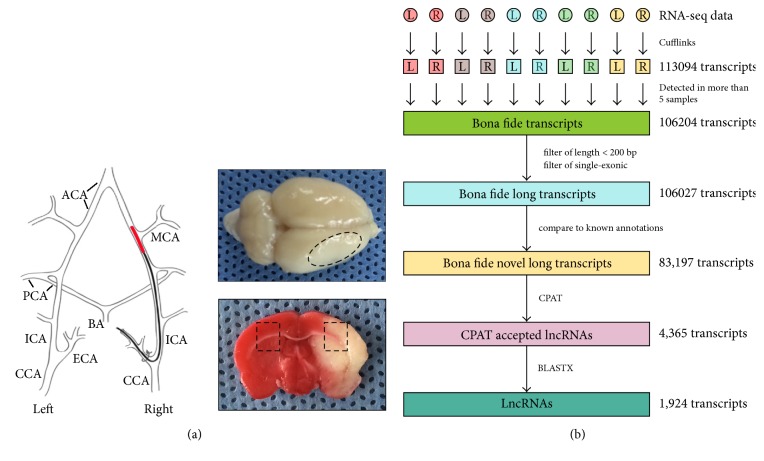
Identification of long noncoding RNAs (lncRNAs) in rat brains. (a) A diagram illustrating MCAO model in rat. Whole brain tissue and TTC stains show infarction in the MCA cortex. Area with dashed line shows the tissue region sampled for RNA sequence and real-time PCR analysis. (b) The pipeline of lncRNAs identification in rat brains.

To obtain a comprehensive view of RNA molecules expressed in ischemic stroke, we examined genome-wide gene expression profiles of rats subjected to MCAO using high throughput sequencing. About 33.7 ± 7 million mapped paired-reads per sample were obtained and we identified 113,094 nonredundant transcripts in ten brain samples (Figure 1(a)).

To identify lncRNAs involved in ischemic stroke, a stringent criterion was applied to eliminate the background noise and errors of nonredundant transcripts. Only those transcripts that were detected in at least five individual samples were identified as bona fide expressed transcripts. On the basis of this analysis, a set of 106,204 bona fide transcripts were defined. After removing single-exon and short transcripts (<200 nt), we obtained 106,027 high-confidence long transcripts. We then removed all transcripts overlapping exons of known genes recorded in NONCODE(v4), NCBI RefSeq, UCSC, and Ensembl databases, resulting in a dataset containing 83,197 high-confidence long transcripts.

Next, CPAT (coding potential assessment tool) was used to calculate the coding potential of high-confidence bona fide novel long transcripts and remove putative protein-coding transcripts. Unlike the large number of annotations available for lncRNAs in human and mouse, there are fewer annotated lncRNAs in rat, so a set of 10,000 rat noncoding intron sequences and a set of 10,000 rat known protein-coding transcripts were used to train the CPAT. As a result, an optimum CPAT score threshold (CPAT score = 0.487) was applied to distinguish noncoding RNA from coding RNA (Figure S1). By CPAT analysis, 4,365 putative long noncoding transcripts were retained. These transcripts were then submitted to BLASTX to scan against the protein database (NR database of NCBI). A set of 1,924 long noncoding transcripts were identified and regarded as novel rat lncRNAs (Figure 1(b) and Table S1).

### 3.2. Genomic Features of LncRNAs in Ischemic Stroke

To further analyze the genomic features of lncRNAs in ischemic stroke, conserved lncRNAs were identified by phyloP score and PhastCons. We found that newly identified lncRNAs are less conserved than protein-coding transcripts, but more conserved than noncoding intron sequences (Figure S2). These features of predicted lncRNAs share similar genomic and evolutionary features with the known rat lncRNAs. The phyloP [22] and PhastCons [23] scores were used to nominate highly conserved and ultraconserved transcripts, respectively. For phyloP score, a cutoff of 0.02638 corresponded to a false discovery rate < 0.01. At this cutoff, the sensitivity for detecting protein-coding transcripts was 0.7488996, and 58 lncRNAs were identified as conserved lncRNAs (Figures S3A and S3B). For contiguous sliding window conservation, a PhastCons score > 0.9805025 corresponded to a false discovery rate < 0.01. At this cutoff, the sensitivity for detecting true positive ultraconserved noncoding element was 0.3452587, and 39 lncRNAs contained ultraconserved elements (Figures S3C and S3D).

### 3.3. Aberrant Expression of LncRNAs in Ischemic Stroke

**Figure 2:**
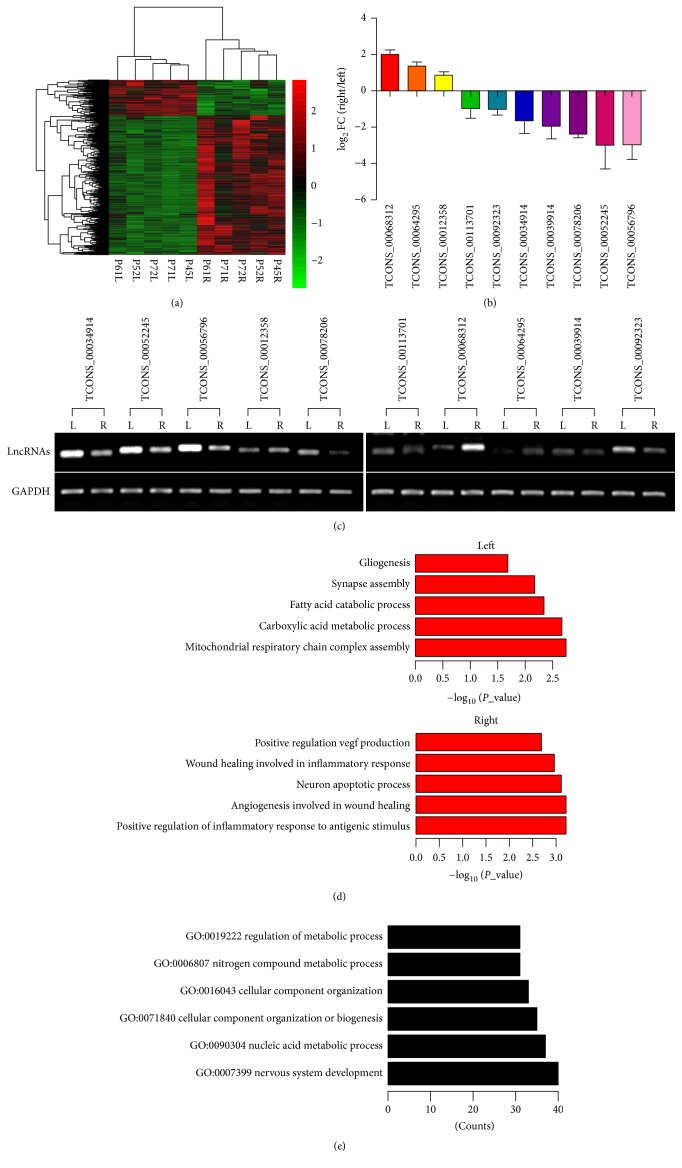
Aberrant expression of lncRNAs in rat brains after ischemic stroke. (a) Heatmap generated from the hierarchical cluster analysis shows the differential expressed genes (lncRNAs, miRNAs, and mRNAs) between ischemia and nonischemia rat brains. Green indicates downregulation and red indicates upregulation. (b) Real-time PCR validation of differential expressed lncRNAs in the ischemia and nonischemia rat brains. (c) Electrophoresis of 10 novel lncRNAs in the ischemia and nonischemia rat brains. (d) KEGG pathway analysis of differential expressed genes in the ischemia and nonischemia rat brains. (e) GO analysis of differential expressed genes in the ischemia and nonischemia rat brains.

To evaluate the effect of ischemic stroke on lncRNA expression profiles, newly identified lncRNAs were combined with Rn5 gene annotation. After transient MCAO, a total of 983 genes including 947 mRNAs and 36 lncRNAs were significantly changed (783 upregulated and 200 downregulated) between the ischemic and paired nonischemic brains after 24 hours of reperfusion. The top 20 most significantly differentially expressed lncRNAs are shown in Table 2. Hierarchical clustering showed systematic variations in the expression of lncRNAs and protein-coding RNAs between ischemic and paired nonischemic brains (Figure 2(a)).

Among the differentially expressed genes in ischemic brains, 36 lncRNAs were deregulated (*P* < 0.05, paired *t*-test), including 10 novel lncRNAs. The existence and dynamic expression patterns of ten novel lncRNAs were validated in another cohort of 10 paired ischemic and nonischemic brains (Figures 2(b) and 2(c)). Many conserved lncRNAs were found among the differentially expressed lncRNAs after ischemic stroke. For example, lncRNA TCONS_00068312 max Phastcon score was 0.807 and was upregulated after ischemic stroke. miR-129-2-3p, which was differently expressed after ischemic stroke, was contained within the intron of TCONS_00068312 (Figure S4). This result indicated that TCONS_00068312-miRNA-129-2-3p might be part of the ischemia response networks which participated in the regulation of ischemic stroke.

### 3.4. Function of LncRNAs in Ischemic Stroke

The upregulated genes were mainly clustered in immune and damage repairing, while the downregulated genes were nervous system and metabolism related genes (Figure 2(d)). In order to better understand the biological functions of lncRNAs, CNC (coding-noncoding) coexpression network between differentially expressed lncRNAs and mRNAs was constructed. We calculate Pearson correlation coefficient between each lncRNAs and mRNAs and chose the highest (top 0.1% of each lncRNAs) and most significantly (*P* < 0.01, Fisher's asymptotic distribution) correlated pairs to construct coexpression network. In this coexpression network, 726 novel lncRNAs were embedded, making it possible to annotate lncRNA functions. The functions of one lncRNA were predicted by analyzing all protein-coding genes that connect to the lncRNA. GO terms annotated in at least 5 genes and with *P* ≤ 0.01 (hypergeometric test) were annotated as potential lncRNA functions. A total of 2602 GO annotations were found and over 334 novel lncRNAs were identified with at least one GO annotation. Biological processes clustered in nervous system development and metabolism were the most significantly dysfunctional GO annotations in ischemic stroke (Figure 2(e)).

Intriguingly, the connected genes of lncRNAs in coexpression network appeared to be involved in a broad range of biological processes, with most of the target genes related to immune and inflammatory response (Figure S5), metabolism and cellular energy (Figure S6), DNA damage and oxidative stress (Figure S7), apoptosis and cell death (Figure S8), angiogenesis and vascular remodeling (Figure S9), and neurogenesis and synaptic plasticity (Figure S10). For example, lncRNA ENSRNOT00000019983 connected to multiple immune and inflammatory response-related genes including ICAM1, Tnfrsf1a, Tnfrsf1b, Nfkb2, Serpine1, and Hmox1 in the CNC coexpression network. All these mRNA transcripts were previously shown to be altered in ischemic stroke [24]. As we observed in the network, one lncRNA could correlate with a large number of target mRNAs, implying that lncRNAs participate in multiple functions in ischemic stroke. Together, these data demonstrate that lncRNAs are aberrantly expressed in ischemic stroke and are involved in the pathophysiology of ischemic stroke.

### 3.5. SMD Network in Ischemic Stroke

**Figure 3:**
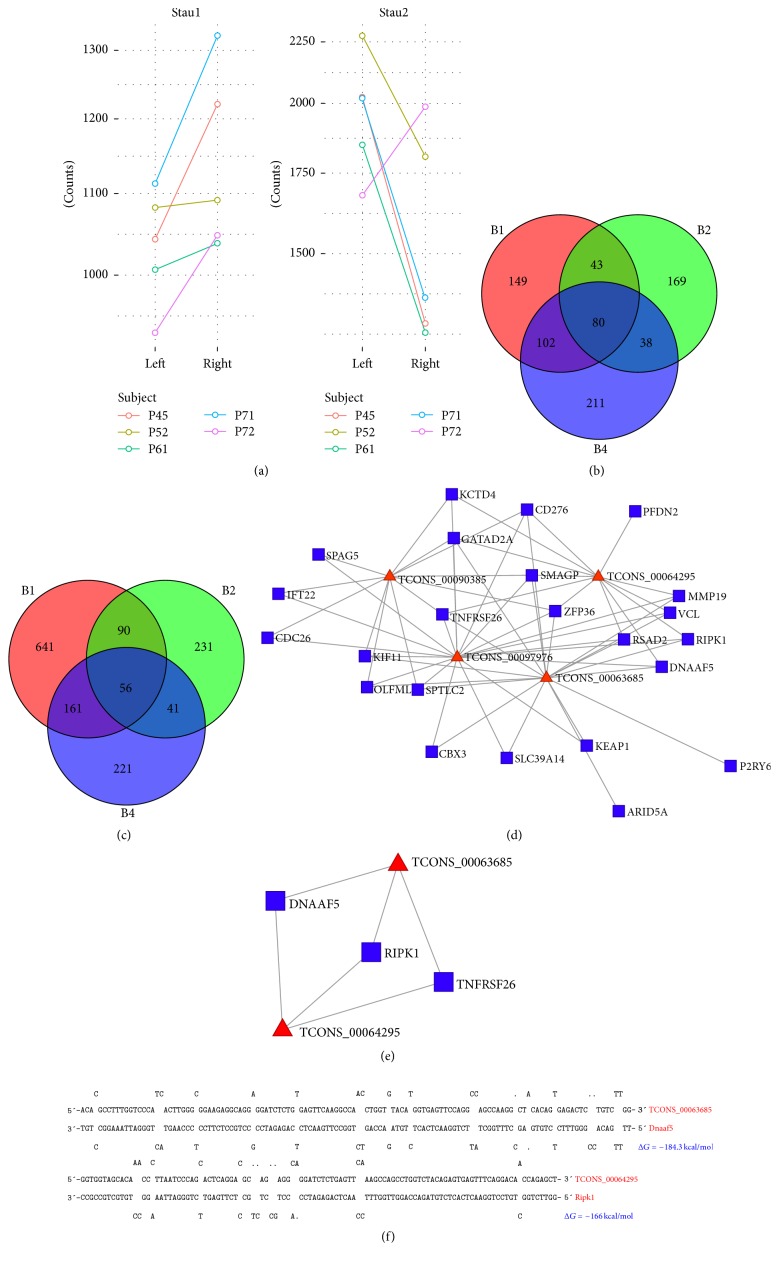
lncRNAs mediate SMD in ischemic stroke. (a) Expression of Staufen1 and Staufen2 in the ischemia and nonischemia rat brain after ischemic stroke. (b) Distribution of SINEs (B1, B2, and B4 element) in mRNA 3′UTR and lncRNAs in the ischemia rat brains. (c) Distribution of SINEs in mRNA 3′UTR and lncRNAs in the nonischemia rat brains. (d) SMD regulatory network of differentially expressed genes. (e) SMD network filtered by duplex energy. (f) Examples of two Alu-based lncRNA:mRNA duplex. Red triangle represents lncRNAs and blue square represents coding genes.

LncRNA may regulate mRNA stability via SMD (Staufen1-mediated mRNA decay) by reverse complement to SINEs (short interspersed nuclear elements) at mRNA 3′UTR. The core effector of SMD, Staufen1 and Staufen2, was changed after ischemic stroke (Figure 3(a)), indicating the role of SMD in stroke. To confirm SINEs mediated SMD in ischemic stroke, distribution of SINEs in rat mRNA 3′UTR and lncRNAs was analyzed in this study. SINEs (B1, B2, and B4 element) were found in 1441 mRNAs (9.32% of total 15446 mRNAs) and 1,135 lncRNAs (39.19% of 2021 new and known lncRNAs) (Figures 3(b) and 3(c)). After thermodynamic duplex analysis of SINEs in lncRNAs and mRNA 3′UTR, 238,061 putative duplexes were formed in 809 mRNAs 3′UTR and 792 lncRNAs. The associated Gibbs free energy of duplex formation (Δ*G*) follows in log-normal distribution (Figures S11–S13). Duplexes with Δ*G* significantly higher than the normal duplex were defined as a putative Staufen1-binding site. A total of 7,970 B1/Alu elements, 4,125 B2 elements, and 4,487 B4 elements were found. Among these genes, only a few are differentially expressed genes (Figures 3(d) and S14). We identified 6 duplexes formed by differentially expressed lncRNAs and mRNAs after ischemic stroke. Interestingly, all the 6 duplexes are B1/Alu SINEs (Figures 3(e) and 3(f)), which is the major family of rat SINE. These data indicate a potential regulatory role of lncRNAs in cerebral ischemia and highlight several SMD candidates dynamically regulated in ischemia.

### 3.6. ceRNA Network in Ischemic Stroke

**Figure 4:**
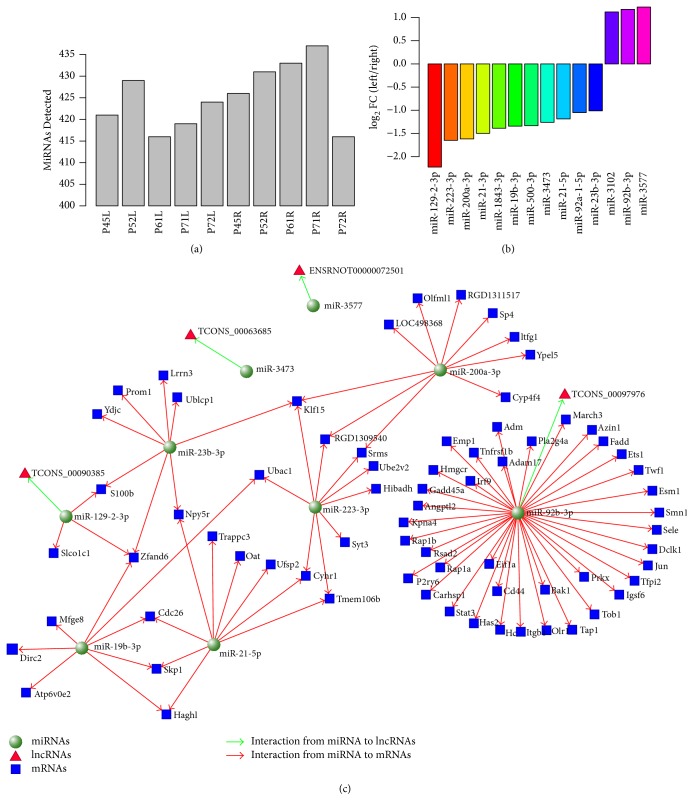
ceRNA network in ischemic stroke. (a) miRNAs detected in the rat brains after ischemic stroke. (b) Expression of top 14 miRNAs in the rat brains after ischemic stroke. (c) mRNA-miRNA-lncRNA interactions network in the ischemic stroke. Triangle represents lncRNAs, square represents coding genes, and circles represent miRNAs. Red lines indicate interaction from miRNA to mRNAs and green lines indicate interaction from miRNA to lncRNAs.

A growing body of evidence supports that lncRNAs act as competitive endogenous RNAs for miRNAs and play roles in physiological and pathological processes. The expression profiles of miRNAs were also analyzed in RNA-seq dataset. A total of 599 miRNAs were identified, which account for 78.3% (599/765) of the rat miRNAs (Figure 4(a)). We found that 14 miRNAs (*P* < 0.05, paired *t*-test; log2 fold change > 1) were differentially expressed between ischemia and paired nonischemia brains after 24 hours of reperfusion (Figure 4(b)). Of these, miR-500-3p, miR-23b-3p, miR-200a-3p, miR-19b-3p, miR-92a-1-5p, miR-21-5p, miR-21-3p, miR-1843-3p, miR-223-3p, miR-3473, and miR-129-2-3p were found to be upregulated, whereas miR-92b-3p, miR-3102, and miR-3577 were found to be downregulated in the rat brain. To establish lncRNA-miRNA interactions, the potential MREs (miRNA Response Elements) in lncRNAs were predicted by miRanda. 42 paired lncRNA-miRNA interactions were identified in 19 differentially expressed lncRNAs, and 4 paired lncRNA-miRNA interactions were left when the negative regulation was taken into consideration. 108 miRNA-mRNA interactions were predicted by miRanda with the same strategy. The miRNA-mRNA interactions were integrated into the coexpression networks. An lncRNA-miRNA-mRNA network which responded to the ischemic stroke was drawn (Figure 4(c)). Intriguingly, miR-129-2-3p and miR-92b-3p were contained in the ceRNA networks. LncRNA TCONS_00097976 is connected to angiogenesis-related miR-92b-3p and angiogenesis-related genes Esm1, Angptl2, and Stat3, which have been reported differently expressed in ischemic stroke [25, 26]. Thus, TCONS_00097976 would compete with miR-92b-3p to regulate these angiogenesis genes during focal ischemia.

## 4. Discussion

A better understanding of the precise molecular mechanisms after cerebral ischemia will be critical for exploring potential new strategies for early diagnosis and therapy of stroke. In this study, we systematically analyzed the lncRNA-involved regulatory networks in rat brain after ischemic stroke based on RNA-seq data of ischemic and nonischemic rat brain tissues. For the first time, our work provides a comprehensive, temporal description of molecular events contributing to the pathogenesis of ischemic stroke and uncovered functional RNAs regulatory networks in ischemic stroke.

In this study, we identified 933 differentially expressed mRNAs which participated in the transcription, metabolism, apoptosis, inflammation, and neuroprotection after ischemic stroke [27, 28]. In line with our results, many genes/proteins such as Hmox1 [24, 29], Gadd45 [30], Ets-1 [31], and Stat3 [4, 32] which are previously shown to be deregulated following stroke were also reported in our study. We observed that several lncRNAs rapidly respond to ischemia, in which 36 lncRNAs are differentially expressed. In support, a previous microarray study showed that cerebral lncRNAs were significantly altered after stroke [6]. In addition, 14 miRNAs were significant altered in the ischemic brains after 24 hours of reperfusion. Some of the differentially expressed miRNAs were previously reported (miR-223, miR-129, and miR-92) [4, 5], which support our results. Nevertheless, many of them had not been reported (miR-500-3p, miR-1843-3p, and miR-3473, miR-3102, and miR-3577). Thus, the results of the present study indicate that focal ischemia significantly alters the temporal expression of many coding and noncoding RNAs, which might be involved in the pathophysiology of stroke.

Although the expression profile of lncRNAs has been shown to change extensively after ischemic stroke, the role of lncRNAs in ischemic stroke is still only partly known. Previous studies found that stroke-induced lncRNAs interacted with chromatin-modifying proteins and modulated ischemic brain damage-related genes [17, 18]. In the present study, the correlations between specific lncRNAs and biological processes of ischemic stroke were connected by the CNC coexpression network. Tnfrsf1a, Tnfrsf1b, and Nfkb2 are known to be involved in inflammatory responses to stroke via noncanonical I-kappa B kinase/NF-kappa B cascade [24]. The inflammatory response, in cooperation with excitotoxic and oxidative responses, is one of the major hazards to ischemic stroke [7]. The high level of coexpression between ENSRNOT00000019983 and immune and inflammatory response gene module indicated that ENSRNOT00000019983 may function as immune and inflammatory response lncRNA in ischemic stroke. We also observed the coexpression between lncRNAs and other modules of genes essential for the pathophysiology of ischemic stroke. Likewise, we identified another five classes of functional lncRNAs which associated with metabolism and cellular energy, DNA damage and oxidative stress, apoptosis and cell death, angiogenesis and vascular remodeling, and neurogenesis and synaptic plasticity in ischemic stroke. These results may serve as a framework for understanding the role of lncRNAs in ischemic stroke. LncRNAs, such as ENSRNOT00000019983, can be therapeutically targeted to minimize poststroke brain damage.

Functional diversity of lncRNAs implicated that lncRNAs might regulate different biological processes through different mechanisms. Staufen1-mediated mRNA decay (SMD) is a translation-dependent mechanism which widely occurs in a number of mammalian cell processes. SINE-containing lncRNAs and SINE-containing mRNA 3′UTRs form intermolecular base-pairing and result in SMD in rat [16]. Previous studies have demonstrated that lncRNAs transactivate SMD by duplexing with 3′UTRs via Alu elements [33]. Brain-specific noncoding RNAs are likely to originate in repeats and repeat elements may play a role in synaptic plasticity [34, 35]. We analyzed the Staufen1-binding site in differently expressed genes and found that several couples of lncRNAs and mRNAs could form a SINE B1/Alu duplex. For example, TCONS_00063685 were found to interact with Dnaa5 (Δ*G* = −184.3 kcal/mol) and TCONS_00063685 interacted with Ripk1 (Δ*G* = −166 kcal/mol). These data imply that lncRNA-mediated SMD was present in ischemic stroke and provide novel insights into lncRNA-based molecular regulatory mechanisms in the pathophysiology of cerebral ischemia. Further studies are needed in the future to show the function of individual lncRNAs in the SMD network after stroke.

ceRNA represents a novel layer of gene regulation that plays important roles in the physiology and development of diseases [15, 36]. It has recently been discovered that pseudogenes, lncRNAs, and circular RNAs act as ceRNA to regulate mRNA expression [37]. In the ceRNA network, lncRNA TCONS_00097976 connected with miR-92b-3p and miR-92b-3p target gene Stat3. Stat3 is a transcription factor which is upregulated after stroke [4, 32]. Previous studies have demonstrated the key angiogenesis functions of Stat3 in ischemic stroke [26, 38]. miR-92 was previously reported as regulator of angiogenesis and cardiac ischemic/reperfusion injury [25, 39]. Thus, TCONS_00097976-miR-92b-3p-Stat3 would be ceRNA mediated ischemia response networks which participated in the regulation of angiogenesis after ischemic stroke. S100b has been reported as a biomarker for ischemic stroke [40, 41]. TCONS_00090385 would compete with miR-129-2-3p and regulated the expression of S100b in ischemic stroke. Hence, TCONS_00090385 and miR-129-2-3p would be potential biomarkers for ischemic stroke.

In conclusion, the present results show that lncRNAs are abnormally expressed after focal ischemia, which suggest that lncRNAs may participate in the pathophysiology of ischemic stroke. Furthermore, extensive bioinformatics analysis revealed several subsets of lncRNAs related to biological processes essential for the pathophysiology of ischemic stroke. Importantly, we have constructed a systematic lncRNA-miRNA-mRNA network which reveals a complex functional noncoding RNA regulatory network in ischemic stroke. Future studies are needed to show whether modulating specific lncRNAs can be a therapeutic option to prevent ischemic pathophysiological events and/or to promote angiogenesis and regeneration.

## Figures and Tables

**Table 1:** Quantitative real-time polymerase chain reaction primer sequences.

Gene	Direction	Primer sequence (5′-3′)
TCONS_00012358	Forward	CACCGGATCACTGAATAGTTCCT
	Reverse	CGATCCGAGCGAAGTAGAATG
TCONS_00056796	Forward	CAATGAATCGCCCAGACTTCTC
	Reverse	GGGACTCATGGCATTAGACATG
TCONS_00068312	Forward	TGACTTCGGTGAGAGCTTTGG
	Reverse	GAGCCTCCGACTTTGGTCTTG
TCONS_00078206	Forward	CTCCTTGAATGTTGGCAGCTAA
	Reverse	GATGTGTGAAGCTGTGAAATGATG
TCONS_00092323	Forward	ACCCTCCTCCACCTACAAATCC
	Reverse	CTGAATGGCCTGGGTTTTATACC
TCONS_00113701	Forward	AACAGGAGGCAAGGCTGTGT
	Reverse	GGCCTTGATCAGCTCATGGT
TCONS_00034914	Forward	ACACAGCCTGCATCGTCACA
	Reverse	CACGACCTTCGAGTCTGCAA
TCONS_00039914	Forward	CTTCCACTGTCTCCCCAATTTATT
	Reverse	TACATTACTCTGCGTCGCCTACA
TCONS_00052245	Forward	GAGCATTGAGTGAAACCAGGAGTT
	Reverse	ATGGAGGCTGAACAAGCGATT
TCONS_00064295	Forward	GTGGAAGCACCAGGAAAGGA
	Reverse	TTAGCCCGATGATGCTCTTGA
rar-GAPDH	Forward	AGTGCCAGCCTCGTCTCATAG
	Reverse	CGTTGAACTTGCCGTGGGTAG

**Table 2:** Top 20 lncRNAs differentially expressed in the rat brain after 24 hours of MCAO.

Test_ID	Gene_symbol	log2Fold_change (left/right)	*P* value	Gene_biotype
TCONS_00063685	TCONS_00063685	0.857714991	0.000144405	Novel Lnc
TCONS_00069225	TCONS_00069225	1.150442621	0.000242217	Novel Lnc
TCONS_00090385	TCONS_00090385	1.245709174	0.001327357	Novel Lnc
TCONS_00084636	TCONS_00084636	−1.03893075	0.004060844	Novel Lnc
TCONS_00072415	TCONS_00072415	−0.355664238	0.002925699	Novel Lnc
TCONS_00068312	TCONS_00068312	−2.118762967	0.001735605	Novel Lnc
TCONS_00097976	TCONS_00097976	−1.202721339	0.000967689	Novel Lnc
TCONS_00064295	TCONS_00064295	−1.904571429	0.000520929	Novel Lnc
TCONS_00013348	TCONS_00013348	−0.516552399	3.90*E* − 05	Novel Lnc
ENSRNOT00000076782	LOC499179	−0.404077908	0.004176249	Processed_pseudogene
ENSRNOT00000011480	Rn50_15_0083.1	−0.38857152	0.002371399	Processed_pseudogene
ENSRNOT00000044465	AABR06026863.1	−0.904025824	0.001450373	Processed_pseudogene
ENSRNOT00000032198	AABR06029867.1	−0.647221245	0.0012401	Processed_pseudogene
ENSRNOT00000049520	AABR06035321.1	−1.129983424	0.001041408	Processed_pseudogene
ENSRNOT00000032154	AABR06005287.1	−1.000000267	0.000777428	Processed_pseudogene
ENSRNOT00000041969	AABR06107883.1	−0.593013236	0.000416013	Processed_pseudogene
ENSRNOT00000067514	Kif1b	−0.638018748	0.001933252	Processed_transcript
ENSRNOT00000076928	Tlr13	−1.506023108	0.001480742	Processed_transcript
ENSRNOT00000076493	Cd93	−2.096878834	0.000644227	Processed_transcript
ENSRNOT00000000787	Arid5b	−0.429661351	0.000163858	Processed_transcript

## Supplementary Materials

Supplementary 1 Table S1: novo lncrna_BMRI_2116963.

Supplementary 2 Figure S1: coding potential assessment tool (CPAT) score of coding and noncoding RNAs in the rat brains.

Supplementary 3 Figure S2: PhastCons score (A) and phyloP score (B) analysis of 10,000 coding transcripts, 10,000 introns, and 1924 novel lncRNAs.

Supplementary 4 Figure S3: (A) fraction of conserved bases of lncRNAs in the rat brains. (B) ROC curve comparing the sensitivity for CPAT coding potential predictions of noncoding RNAs versus protein-coding genes. (C) Maximum 200 nt window of lncRNAs in the rat brains. (D) ROC curve for predicting ultraconserved noncoding elements versus random intergenic controls.

Supplementary 5 Figure S4: miR-129-2-3p located in the intron of lncRNA TCONS_00068312.

Supplementary 6 Figure S5: immune and inflammatory response-related lncRNAs in ischemic stroke. Triangles represent lncRNAs, and circles represent coding genes. Red lines indicate negative correlations and green lines indicate positive correlations.

Supplementary 7 Figure S6: metabolism and cellular energy related lncRNAs in ischemic stroke. Triangles represent lncRNAs, and circles represent coding genes. Red lines indicate negative correlations and green lines indicate positive correlations.

Supplementary 8 Figure S7: DNA damage and oxidative stress related lncRNAs in ischemic stroke. Triangles represent lncRNAs, and circles represent coding genes. Red lines indicate negative correlations and green lines indicate positive correlations.

Supplementary 9 Figure S8: apoptosis and cell death related lncRNAs in ischemic stroke. Triangles represent lncRNAs, and circles represent coding genes. Red lines indicate negative correlations and green lines indicate positive correlations.

Supplementary 10 Figure S9: angiogenesis and vascular remodeling related lncRNAs in ischemic stroke. Triangles represent lncRNAs, and circles represent coding genes. Red lines indicate negative correlations and green lines indicate positive correlations.

Supplementary 11 Figure S10: neurogenesis and synaptic plasticity related lncRNAs in ischemic stroke. Triangles represent lncRNAs, and circles represent coding genes. Red lines indicate negative correlations and green lines indicate positive correlations.

Supplementary 12 Figure S11: Δ*G* distribution of B2 duplex between mRNA 3′UTR and lncRNAs in ischemic stroke.

Supplementary 13 Figure S12: Δ*G* distribution of Alu duplex between mRNA 3′UTR and lncRNAs in ischemic stroke.

Supplementary 14 Figure S13: Δ*G* distribution of B4 duplex between mRNA 3′UTR and lncRNAs in ischemic stroke.

Supplementary 15 Figure S14: (A) SMD regulatory network of B2 differentially expressed genes. (B) SMD regulatory network of B4 differentially expressed genes.
