# Supplementary figures and images for: Systematic Analysis of RNA Regulatory Network in Rat Brain after Ischemic Stroke
Source: Biomed Res Int. 2018 Jan 8;2018:8354350. doi: 10.1155/2018/8354350 (PMC5817225; doi:10.1155/2018/8354350)

A

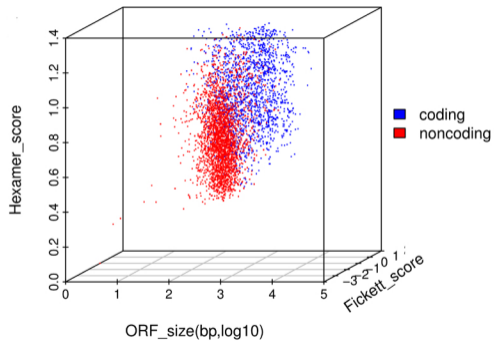

B

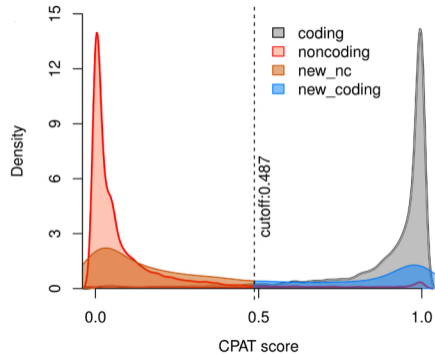

Supplement: Supplementary 2 — Figure S1: coding potential assessment tool (CPAT) score of coding and noncoding RNAs in the rat brains. [file 8354350.f2.pdf]

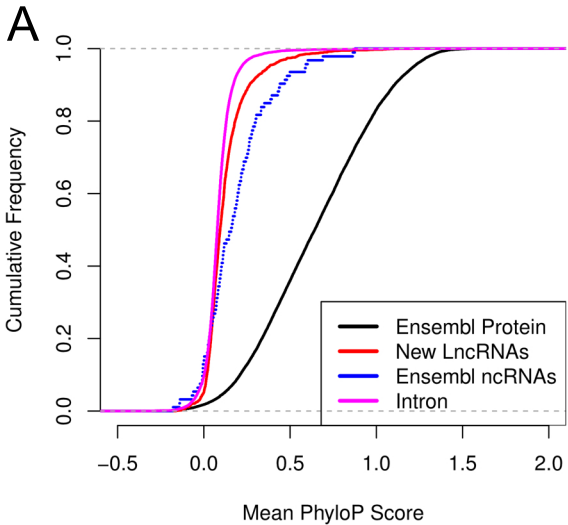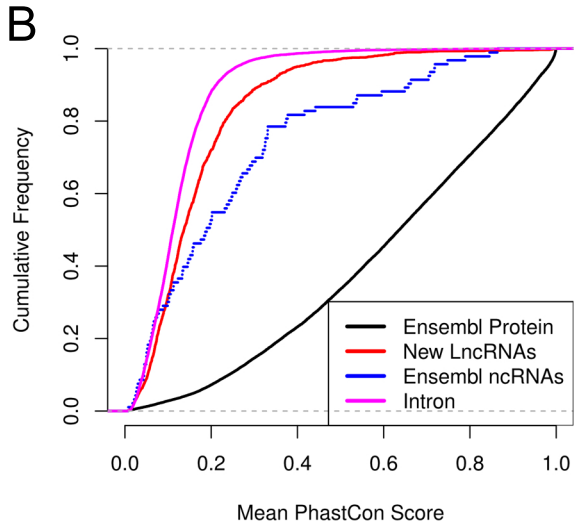

Supplement: Supplementary 3 — Figure S2: PhastCons score (A) and phyloP score (B) analysis of 10,000 coding transcripts, 10,000 introns, and 1924 novel lncRNAs. [file 8354350.f3.pdf]

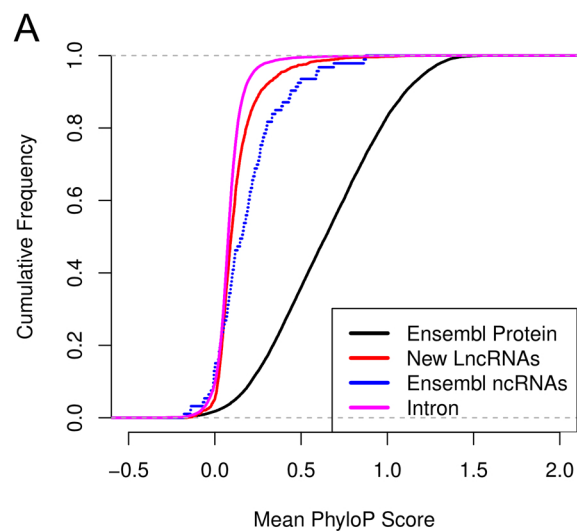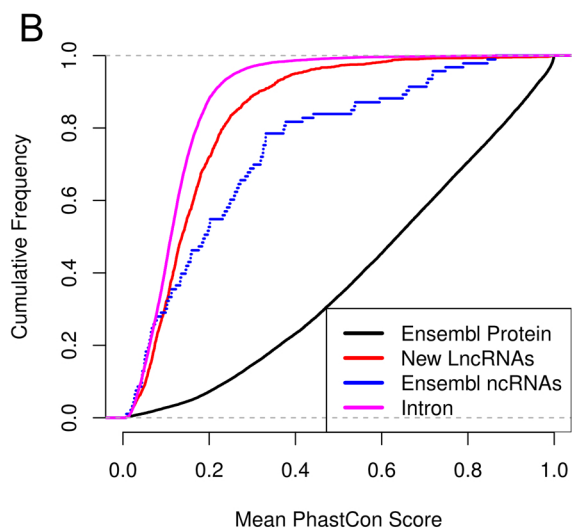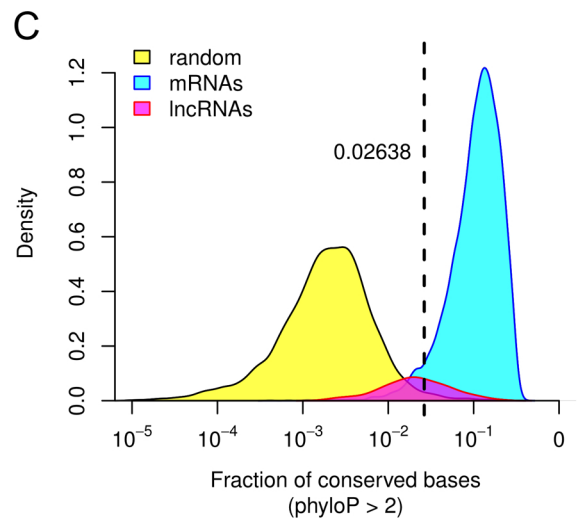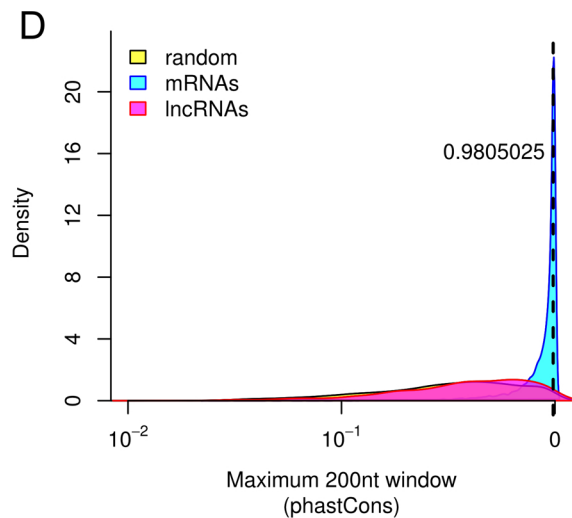

Supplement: Supplementary 4 — Figure S3: (A) fraction of conserved bases of lncRNAs in the rat brains. (B) ROC curve comparing the sensitivity for CPAT coding potential predictions of noncoding RNAs versus protein-coding genes. (C) Maximum 200 nt window of lncRNAs in the rat brains. (D) ROC curve for predicting ultraconserved noncoding elements versus random intergenic controls. [file 8354350.f4.pdf]

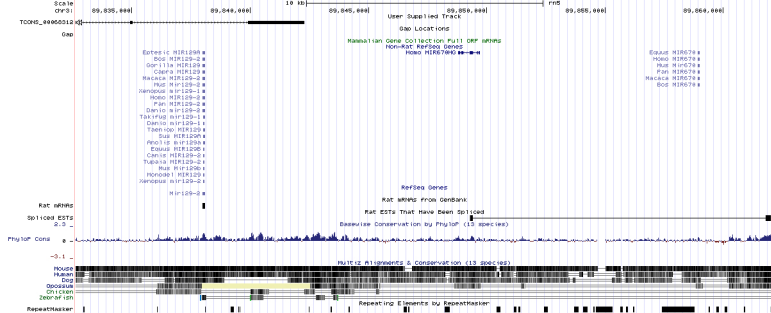

Supplement: Supplementary 5 — Figure S4: miR-129-2-3p located in the intron of lncRNA TCONS_00068312. [file 8354350.f5.pdf]

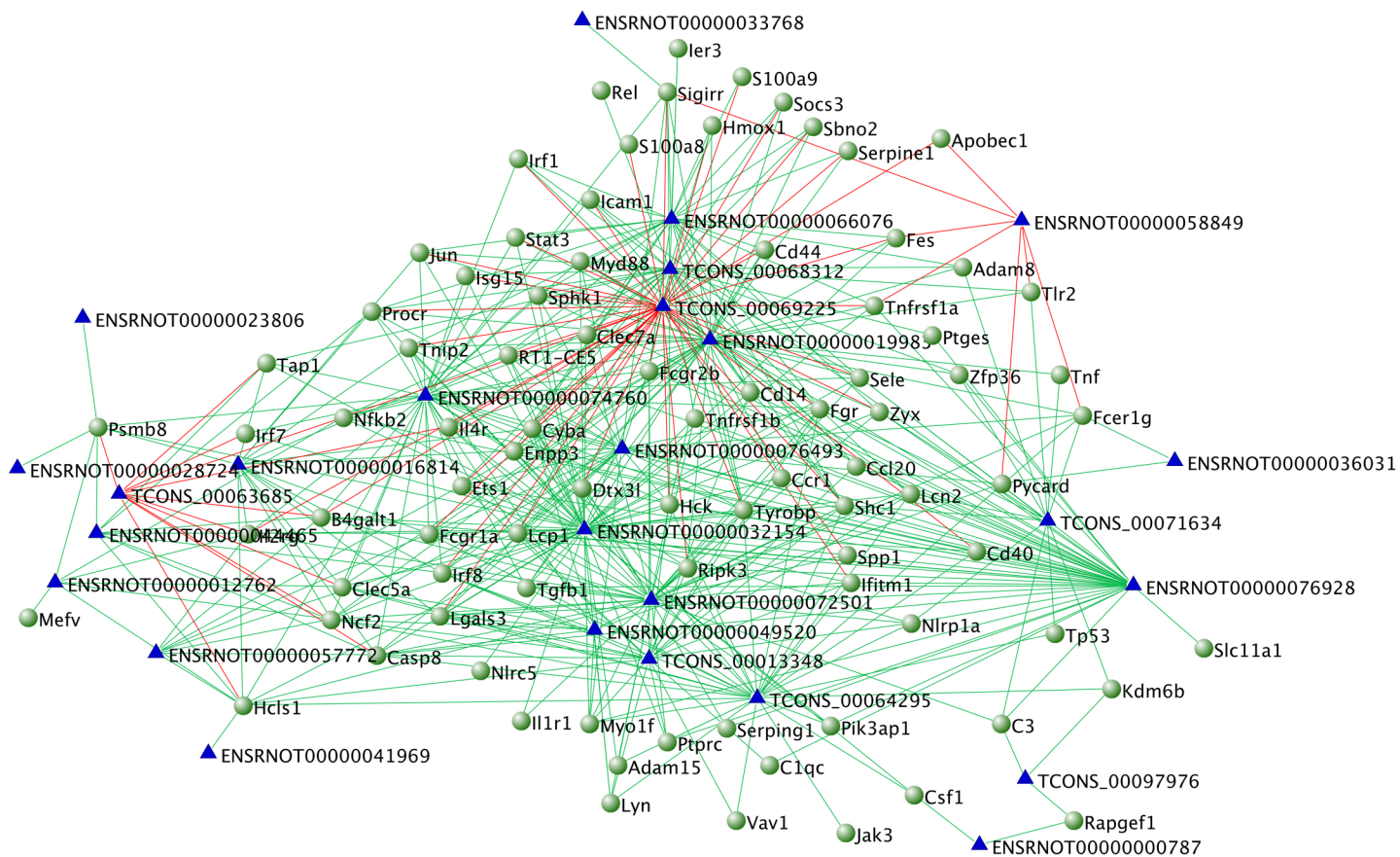

Supplement: Supplementary 6 — Figure S5: immune and inflammatory response-related lncRNAs in ischemic stroke. Triangles represent lncRNAs, and circles represent coding genes. Red lines indicate negative correlations and green lines indicate positive correlations. [file 8354350.f6.pdf]

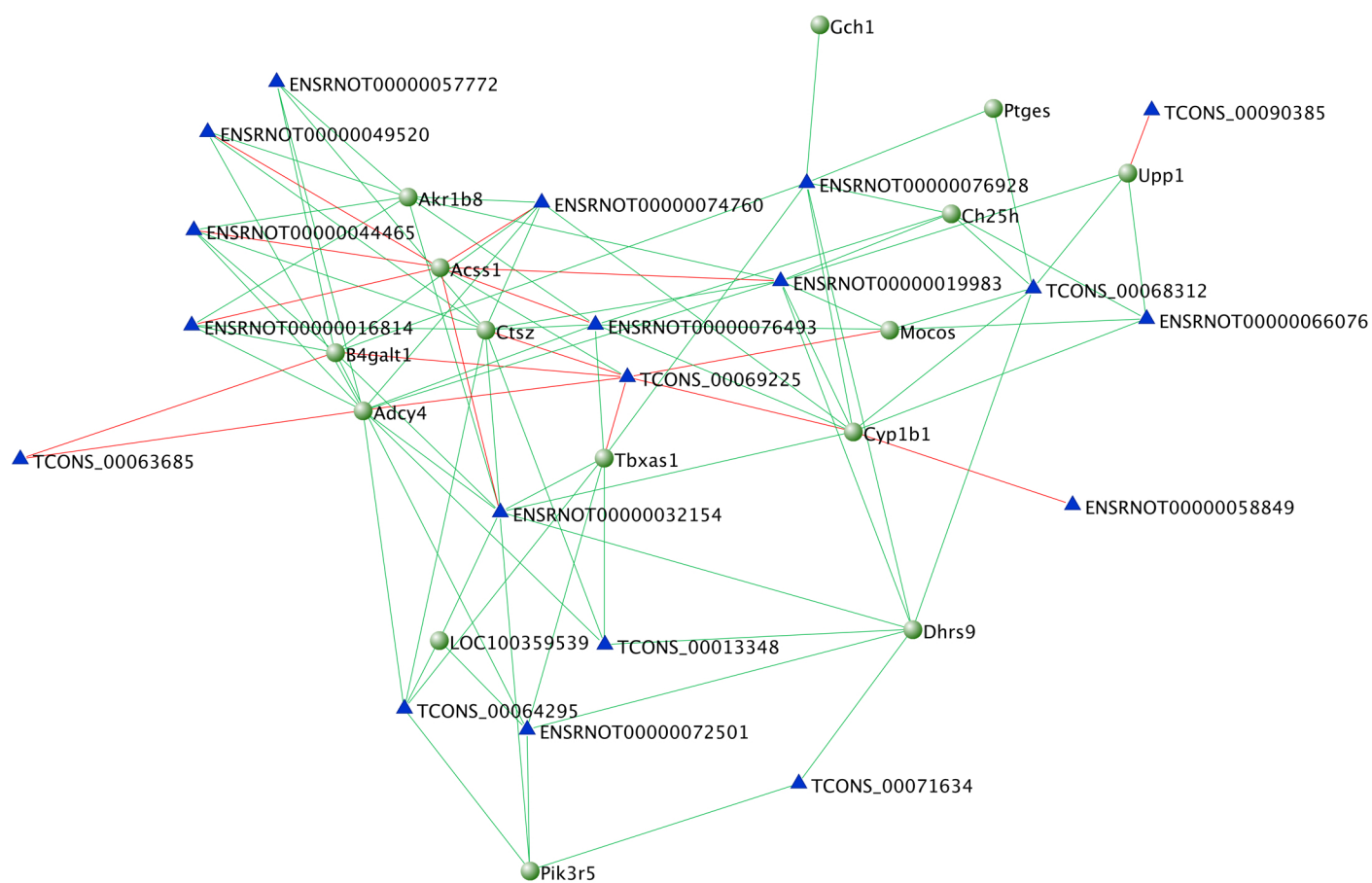

Supplement: Supplementary 7 — Figure S6: metabolism and cellular energy related lncRNAs in ischemic stroke. Triangles represent lncRNAs, and circles represent coding genes. Red lines indicate negative correlations and green lines indicate positive correlations. [file 8354350.f7.pdf]

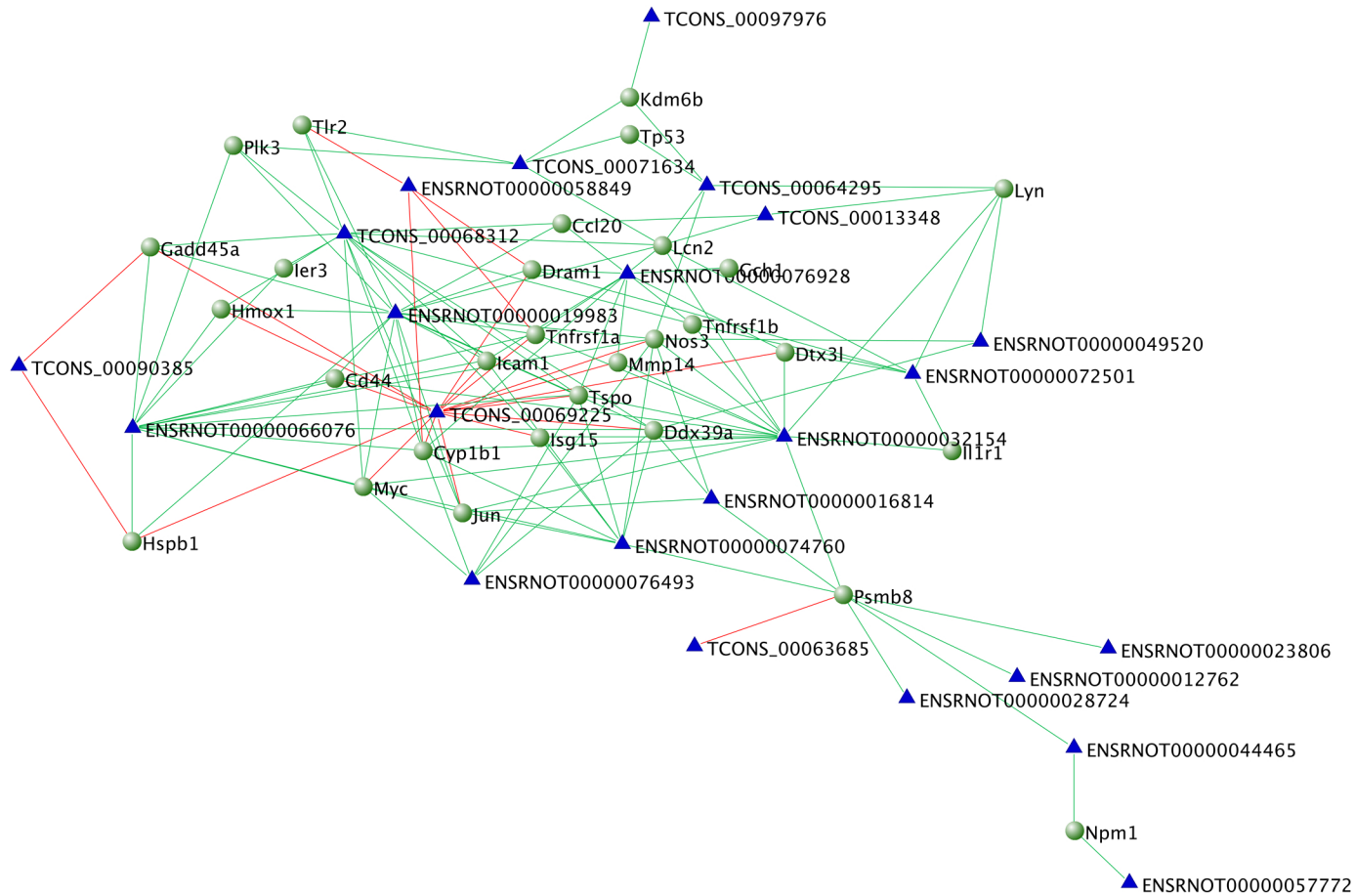

Supplement: Supplementary 8 — Figure S7: DNA damage and oxidative stress related lncRNAs in ischemic stroke. Triangles represent lncRNAs, and circles represent coding genes. Red lines indicate negative correlations and green lines indicate positive correlations. [file 8354350.f8.pdf]

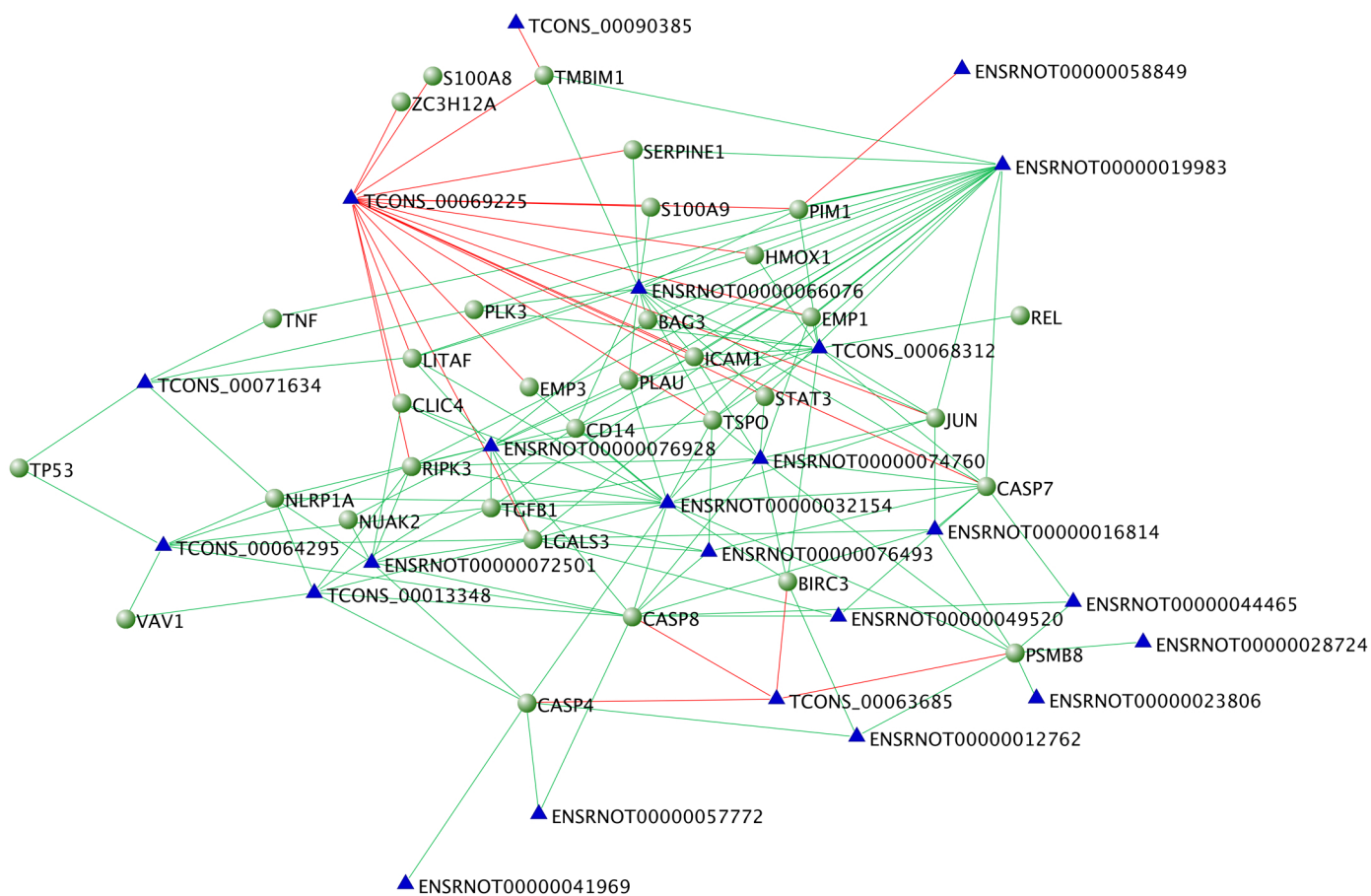

Supplement: Supplementary 9 — Figure S8: apoptosis and cell death related lncRNAs in ischemic stroke. Triangles represent lncRNAs, and circles represent coding genes. Red lines indicate negative correlations and green lines indicate positive correlations. [file 8354350.f9.pdf]

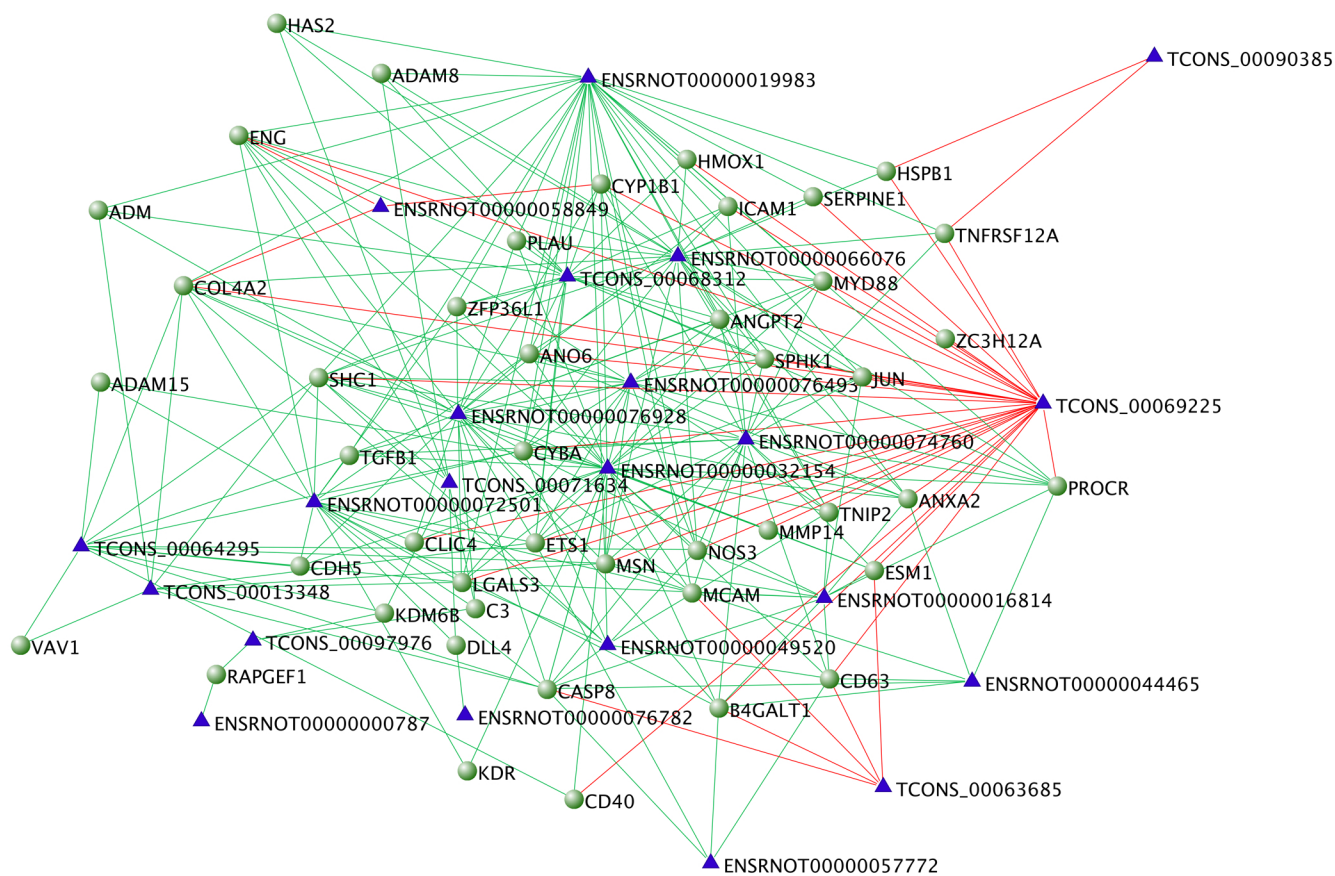

Supplement: Supplementary 10 — Figure S9: angiogenesis and vascular remodeling related lncRNAs in ischemic stroke. Triangles represent lncRNAs, and circles represent coding genes. Red lines indicate negative correlations and green lines indicate positive correlations. [file 8354350.f10.pdf]

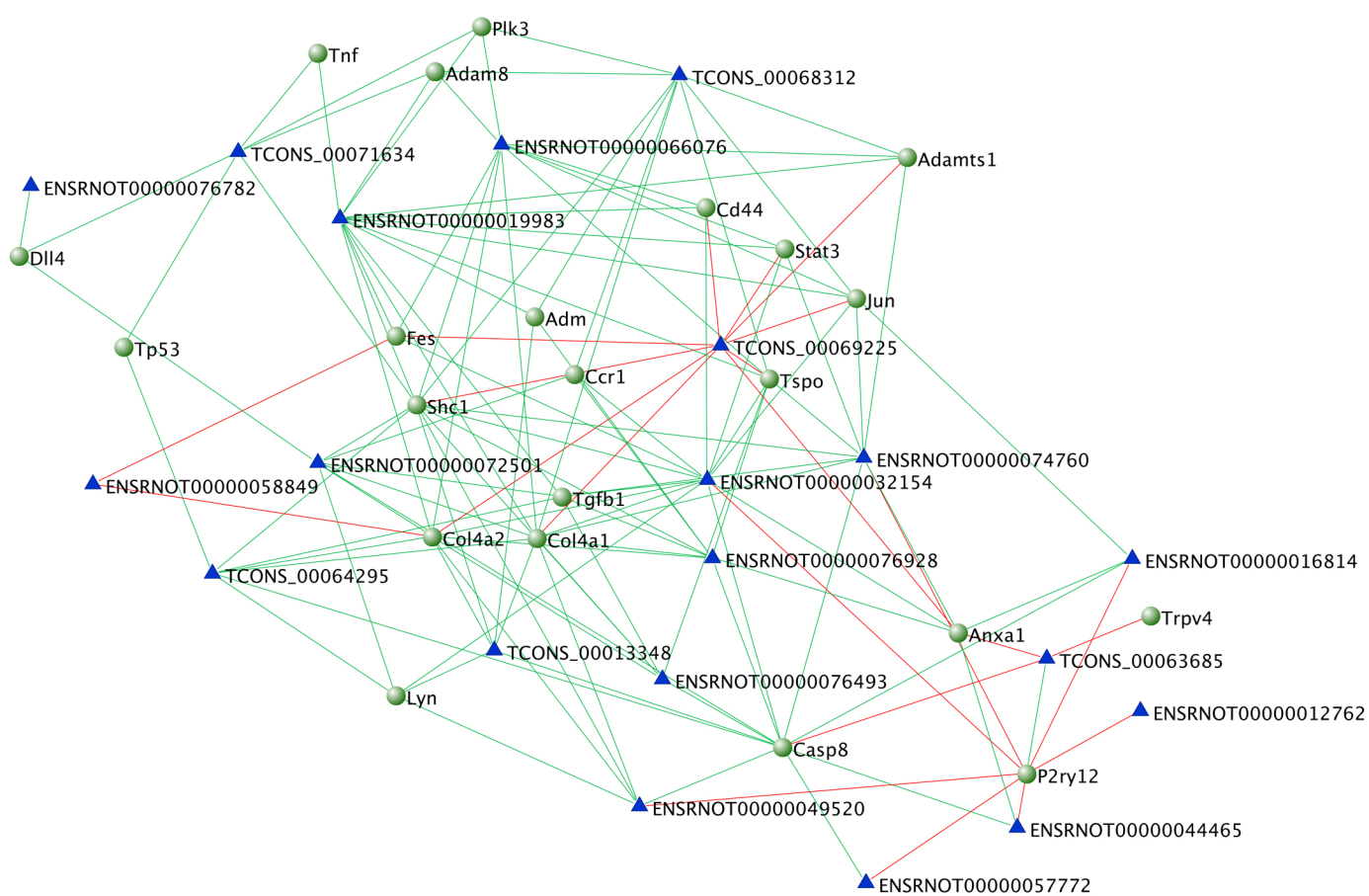

Supplement: Supplementary 11 — Figure S10: neurogenesis and synaptic plasticity related lncRNAs in ischemic stroke. Triangles represent lncRNAs, and circles represent coding genes. Red lines indicate negative correlations and green lines indicate positive correlations. [file 8354350.f11.pdf]

### Empirical and theoretical den

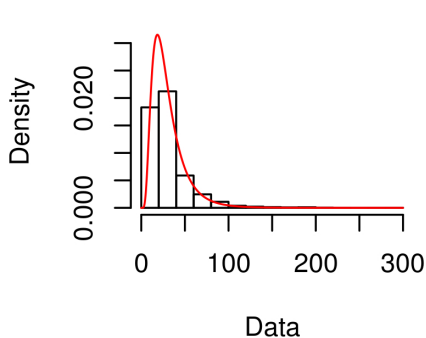

### Q-Q plot

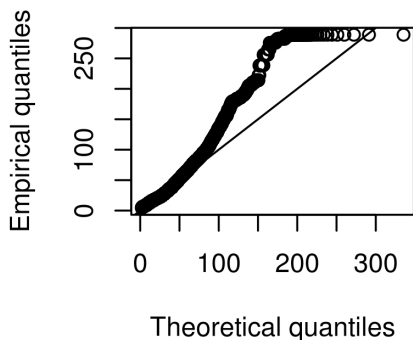

### Empirical and theoretical CDF

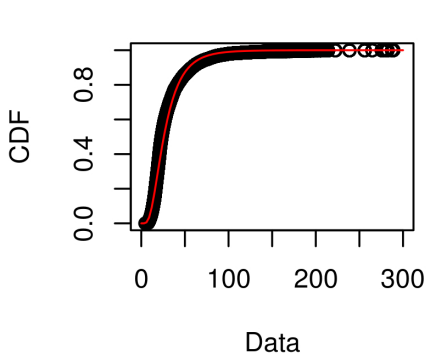

### P-P plot

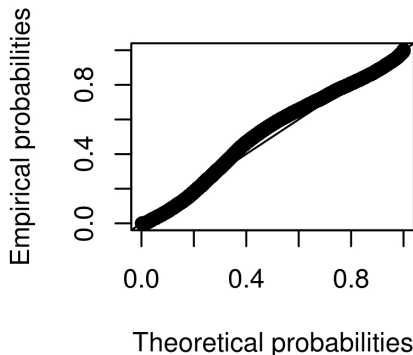

Supplement: Supplementary 12 — Figure S11: ΔG distribution of B2 duplex between mRNA 3′UTR and lncRNAs in ischemic stroke. [file 8354350.f12.pdf]

### Empirical and theoretical den

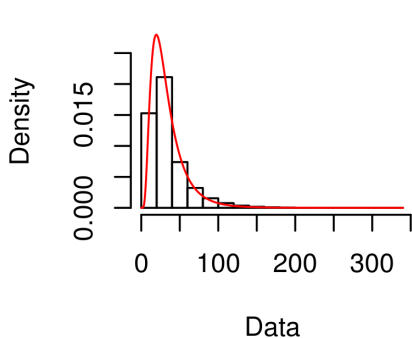

### Q-Q plot

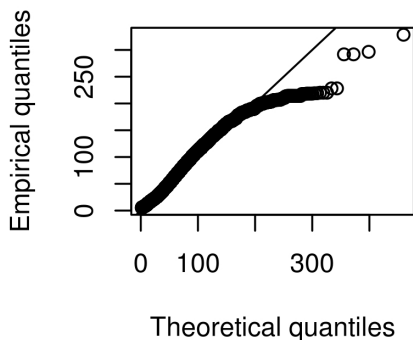

### Empirical and theoretical CDF

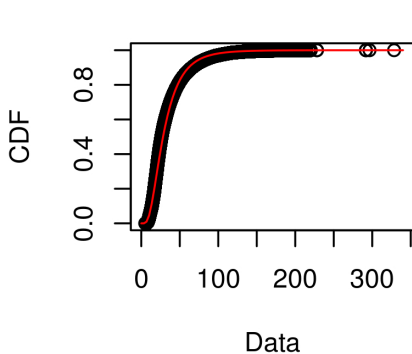

### P-P plot

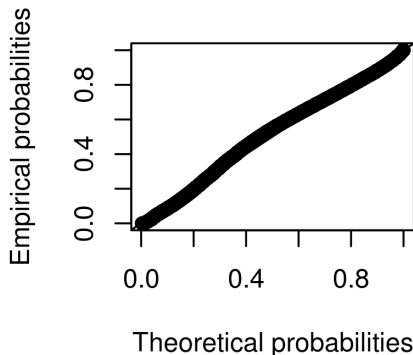

Supplement: Supplementary 13 — Figure S12: ΔG distribution of Alu duplex between mRNA 3′UTR and lncRNAs in ischemic stroke. [file 8354350.f13.pdf]

### Empirical and theoretical den

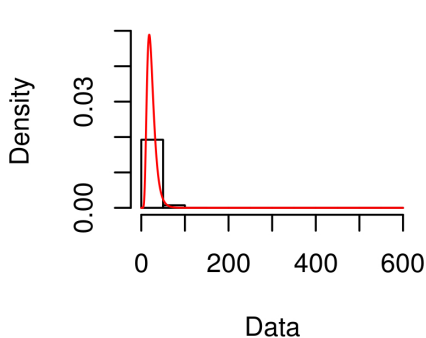

### Q-Q plot

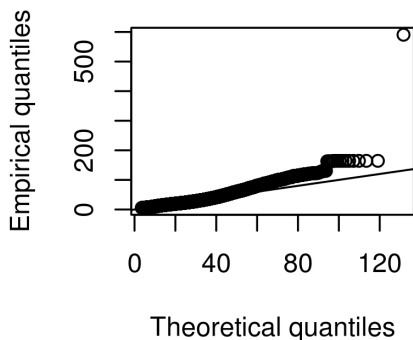

### Empirical and theoretical CDF

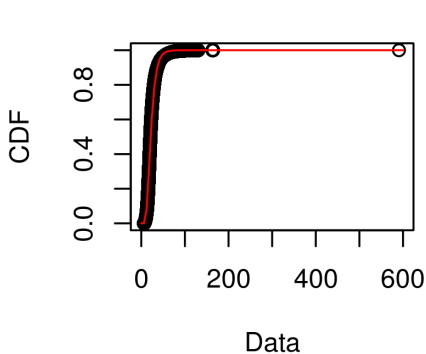

### P-P plot

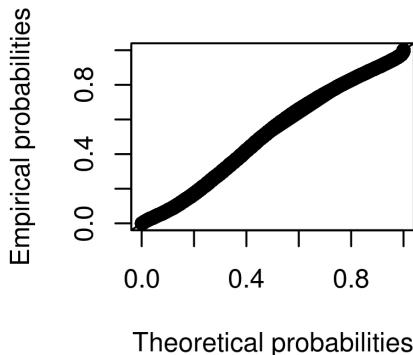

Supplement: Supplementary 14 — Figure S13: ΔG distribution of B4 duplex between mRNA 3′UTR and lncRNAs in ischemic stroke. [file 8354350.f14.pdf]

A

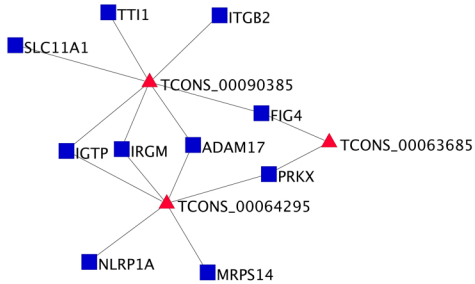

B

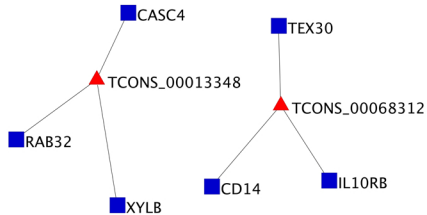

Supplement: Supplementary 15 — Figure S14: (A) SMD regulatory network of B2 differentially expressed genes. (B) SMD regulatory network of B4 differentially expressed genes. [file 8354350.f15.pdf]
